# X-ray diffraction from flight muscle with a headless myosin mutation: implications for interpreting reflection patterns

**DOI:** 10.3389/fphys.2014.00416

**Published:** 2014-10-29

**Authors:** Hiroyuki Iwamoto, Károly Trombitás, Naoto Yagi, Jennifer A. Suggs, Sanford I. Bernstein

**Affiliations:** ^1^Research and Utilization Division, Japan Synchrotron Radiation Research Institute, SPring-8Hyogo, Japan; ^2^Veterinary and Comparative Anatomy, Pharmacology and Physiology, Washington State UniversityPullman, WA, USA; ^3^Department of Biology, Molecular Biology Institute, Heart Institute, San Diego State UniversitySan Diego, CA, USA

**Keywords:** myosin mutation, insect flight muscle, synchrotron radiation, X-ray diffraction, *Drosophila*, electron microscopy

## Abstract

Fruit fly (*Drosophila melanogaster*) is one of the most useful animal models to study the causes and effects of hereditary diseases because of its rich genetic resources. It is especially suitable for studying myopathies caused by myosin mutations, because specific mutations can be induced to the flight muscle-specific myosin isoform, while leaving other isoforms intact. Here we describe an X-ray-diffraction-based method to evaluate the structural effects of mutations in contractile proteins in *Drosophila* indirect flight muscle. Specifically, we describe the effect of the headless myosin mutation, *Mhc*^*10*^-*Y97*, in which the motor domain of the myosin head is deleted, on the X-ray diffraction pattern. The loss of general integrity of the filament lattice is evident from the pattern. A striking observation, however, is the prominent meridional reflection at *d* = 14.5 nm, a hallmark for the regularity of the myosin-containing thick filament. This reflection has long been considered to arise mainly from the myosin head, but taking the 6th actin layer line reflection as an internal control, the 14.5-nm reflection is even stronger than that of wild-type muscle. We confirmed these results via electron microscopy, wherein image analysis revealed structures with a similar periodicity. These observations have major implications on the interpretation of myosin-based reflections.

## Introduction

Despite the long phylogenic distance between humans and insects, striking similarities exist in the structure of their muscles. Both the skeletal or cardiac muscles of vertebrates and somatic muscles of insects are cross-striated, have similar sarcomeric structure and are regulated by the thin-filament-based system involving troponin and tropomyosin. In this respect, *Drosophila melanogaster* is one of the best-suited model animals to study congenital myopathies that occur in humans, owing to its rich genetic resources and ease of breeding. Its indirect flight muscle is the preferred target, because it is the bulkiest of all muscles in the insect, and many of its constituent contractile proteins, including myosin and actin, are expressed as tissue-specific isoforms (Bernstein et al., 1993). Therefore, the mutations or knock-outs of these isoforms are usually viable because non-flight muscle isoforms remain intact.

Here we evaluate the structural consequences of the headless mutant of flight muscle myosin isoform, in which an adult myosin rod transgene (*Y97*) is expressed in *Mhc^*10*^* myosin-null background (Cripps et al., 1999). All of the motor domain and a part of the binding site for the essential light chain are missing from the rod molecule, but the binding site for the regulatory light chain remains intact (Cripps et al., 1999). Electron microscopy of the flight muscle in this mutant has shown that the rod molecules form thick filaments with hollow centers as in wild type, and that these thick filaments are often surrounded by thin filaments and form a hexagonal lattice, but the integrity of the whole myofibrils is inferior to that in wild type (Cripps et al., 1999).

We adopt another approach to evaluate the structure of the mutated flight muscle, i.e., X-ray diffraction. The technique of X-ray diffraction is especially suitable for reporting the regularity of arrangement of contractile proteins in myofilaments, and also the spatial arrangement of myofilaments within a sarcomere. The basic knowledge about X-ray diffraction from muscle was initially established by using bulky isolated whole muscles from frog (e.g., Huxley and Brown, 1967), but brighter X-ray sources, including synchrotron radiation facilities, and more advanced sensitive detectors have made it possible to record X-ray diffraction patterns from wider varieties of muscle specimens with much shorter exposure times. Compared with other techniques, X-ray diffraction can be applied to samples under physiological conditions, and time-resolved measurements are possible (for more detailed explanations for the synchrotron-based X-ray diffraction technique and comparisons with other techniques, refer to Oiwa et al., 2009). In fact, the X-ray diffraction technique has been applied to biopsied specimens of human muscle with congenital myopathies (Ochala et al., 2010; Ochala and Iwamoto, 2013), specimens from transgenic mice with expressed mutations found in human myopathy patients (Ochala et al., 2011, 2013, 2014; Lindqvist et al., 2012, 2013) and rat disease models (Corpeno et al., 2014). The intense and well-oriented X-rays from the third-generation synchrotron radiation facilities have made it possible to record full 2-D diffraction patterns of *Drosophila* indirect flight muscles (Irving and Maughan, 2000; Dickinson et al., 2005; Iwamoto et al., 2007).

Here we tested several newly developed techniques to record X-ray diffraction patterns from glycerinated *Drosophila* flight muscle specimens, which are too small for ordinary mounting techniques for longer muscle fibers, and the qualities of the obtained diffraction patterns were compared. We applied these techniques combined with electron microscopy analysis to the *Mhc^*10*^-Y97* mutant. This mutant is especially suited for studying the role of the myosin motor domain on the general architecture of sarcomeres, and its contributions to the intensities of the myosin-based reflections. The unexpected findings presented here lead to important implications for the interpretation of muscle X-ray diffraction in general.

## Materials and methods

### Specimen

The strains *Mhc^*10*^* (homozygous myosin null in the indirect flight muscles; Collier et al., 1990) and *Mhc^*10*^-Y97* (homozygous myosin null in the indirect flight muscles and homozygous for a transgene expressing headless myosin specifically in these muscles; Cripps et al., 1999) of *D. melanogaster* were verified as to myosin protein content and maintained at San Diego State University. Flies were transferred to SPring-8, and were maintained there for X-ray diffraction studies. The wild type of *D. melanogaster* (Hikone-R strain) was obtained from Ehime University (Iwamoto et al., 2007). Insects other than *Drosophila* were collected at the campus of SPring-8.

For X-ray diffraction studies, the whole thoraces of *Drosophila* adults were glycerinated in a 50% mixture of glycerol and a relaxing solution (for composition see Iwamoto, 2009; Iwamoto et al., 2010) containing phenylmethylsulfoxide and protease inhibitor cocktail (P8340, Sigma-Aldrich, St. Louis, USA), and stored at -20°C in a freezer. The flight muscles of other insects were treated in a similar way. The methods of mounting of *Drosophila* specimens for X-ray recording are detailed in the Results section. Flight muscle fibers of larger insects were mounted as described (Iwamoto et al., 2001, 2010; Iwamoto, 2009).

### X-ray diffraction recordings

X-ray diffraction patterns of flight muscle were recorded at the BL45XU small-angle scattering beamline of SPring-8 (Fujisawa et al., 2000). Details of the methods of recording have been described (Iwamoto et al., 2001, 2003; Iwamoto, 2009). Briefly, the specimens were placed in a rigor solution with a composition described previously (Iwamoto, 2000), except for samples from horsefly, which were placed in a relaxing solution (Iwamoto, 2009). In addition, the solution contained 5 mM dithiothreitol to reduce radiation damage. Monochromatized X-ray beams (wavelength, 0.09 or 0.1 nm; flux, 10^12^ photons/s, beam size, 0.3 × 0.2 mm) were irradiated to the specimen, and the patterns were recorded by using a cooled CCD camera (C4880-50, Hamamatsu Photonics, Hamamatsu, Japan) in combination with an image intensifier (VP5445MOD, Hamamatsu Photonics). The time for single exposure was 1 s. A maximum of 20 exposures were recorded from a single specimen, and the patterns were summed. The background scattering was subtracted by the method described in Iwamoto et al. (2003, 2010).

### Electron microscopy

Indirect flight muscle bundles for electron microscopic observations were dissected at San Diego State University from 2-day-old flies in relaxing solution (Peckham et al., 1990) containing 1% Triton X-100 on ice. After 3 h, the liquid was replaced successively by two washes of fresh relaxing solution containing 50% glycerol and a dissolved Roche protease inhibitor cocktail tablet (aprotinin, leupeptin, EDTA, pefablock). Muscles were stored at −20°C and shipped to Washington State University on ice, where they were fixed with 3% glutaraldehyde, then postfixed with 1% osmium tetroxide in 100 mM phosphate buffer, 10 mM MgC1_2_; pH 6 (Trombitas and Pollack, 1995). Samples were embedded in Araldite 506. Ultrathin sections (~20 nm) were cut with an LKB Ultrotome III. Sections were stained with potassium permanganate, and lead citrate, and observed and photographed with a Philips 420 electron microscope.

## Results

### Mounting techniques for drosophila flight muscle

The length of the indirect flight muscle fibers of *D. melanogaster* is only ~1 mm, and it is very difficult to mount both ends with metal foil clips, as would often be done with longer specimens, while keeping the myofibrils straight (this is required to yield good diffraction patterns). 2-D diffraction patterns from *Drosophila* flight muscles have been recorded from whole live insects by using two configurations.

**Figure 1:**
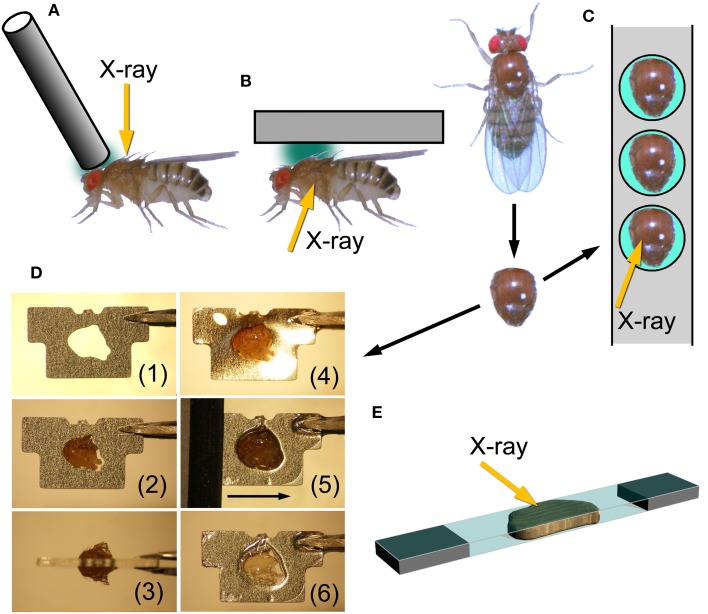
**Mounting techniques for *Drosophila* indirect flight muscle**. **(A,B)** Methods to record X-ray diffraction patterns from live flies; **(C–E)** methods to record patterns from glycerinated muscle preparations. **(A)**, attaching a metal wire on top of the head and at the anterior end of the thorax and the fly is irradiated with X-rays from the top. This is the method adopted by Irving and Maughan (2000), Dickinson et al. (2005). **(B)**, a fly, glued to a substrate, is irradiated from the side to record patterns from both of the two antagonistic flight muscles (DLM and DVM). This is the configuration used by Iwamoto et al. (2007). **(C)**, gluing an isolated thorax to a stainless-steel plate with round holes. After gluing, unwanted tissues, such as leg muscles and digestive tract, are removed. (**D**), procedure to glue an isolated thorax to a stainless-steel plate with a precision-etched hole and to remove excess parts of the thorax. (1), a plate before mounting; (2) an isolated thorax fitted to the hole; (3) top view; (4) thorax glued to the plate, (5) an ultrasonic-vibrated microtome blade slides along the surface of the plate; (6) finished sample with exposed DLM. **(E)**, sandwiching an isolated flight muscle between two thin plastic films (1.5 μm-thick polyester, Chemplex Industries, Palm City, USA). Incident X-ray beams make a small angle with respect to the film plane.

One is to attach a hollow tube on top of the thorax and irradiate X-rays through it (Irving and Maughan, 2000; Dickinson et al., 2005). By doing so one can obtain diffraction patterns from the dorsal longitudinal muscle (DLM) alone, one of the two antagonistic indirect flight muscles (Figure 1A). The other is to glue the whole insect, and irradiate X-rays from the side (Figure 1B; Iwamoto et al., 2007), similar to the configuration used for recording diffraction patterns from live bumblebees (Iwamoto and Yagi, 2013). In this configuration, one can simultaneously record diffraction patterns from both of the two antagonistic flight muscles (DLM and dorsoventral muscle, DVM) as well as the jump muscle. In Iwamoto et al. (2007) this configuration was used also for recording diffraction patterns from pupae to study the time course of development of flight muscle architecture.

The drawbacks of such *in vivo* X-ray recording are that (1) there are unwanted materials in the beampath (such as legs, leg muscles, nerves, digestive tracts, etc.) that could compromise the quality of diffraction patterns, and that (2) the solution environment of contractile proteins cannot be changed at will. For these reasons it is desirable to establish techniques to record diffraction patterns from demembranated (glycerinated) flight muscle specimens from *Drosophila*.

Here we tested several techniques to record diffraction patterns from glycerinated specimens, besides the conventional clamping technique. The first one is to use a stainless-steel plate with round holes. The glycerinated thorax was placed in the hole and glued by using a cyanoacrilic resin (Figure 1C). After gluing, the legs and other unwanted materials were removed. By irradiating X-rays in the middle of the hole, one can obtain diffraction patterns from DLM alone. The DLM fibers were irradiated from the top of the thorax.

The second technique tested was to fabricate stainless-steel plates (thickness, 0.2 mm) by photo-etching, each with a hole shaped to fit the contour of the thorax. In this method, the orientation of DLM fibers can be precisely fixed with respect to the stainless-steel plate. The thorax was glued so that equal amounts of volume of the thorax stick out of both sides of the plate. After this, the parts of the thorax sticking out of the plate were cut off by using an ultrasonic vibration cutter with microtome blades. This leaves DLM fibers within the thickness of the plate. X-rays were irradiated from the side (Figure 1D).

The third technique was to isolate the assembly of 6 indirect flight muscle fibers (both DLM and DVM have 6 flight muscle fibers on each side), and sandwich them with two very thin plastic films (Figure 1E). The plastic films had been glued to two pieces of thin plastic plate at both ends, leaving a small gap between them so that the fibers were not crushed. X-ray beams were irradiated so that the beam axis made a small angle with respect to the film plane. By doing so one should be able to eliminate the parasitic scattering resulting from the total reflection from the film surface. However, the fact was that some parasitic scattering was recorded, probably because the film surface was somewhat wavy.

### Diffraction patterns recorded from wild-type drosophila flight muscle

**Figure 2:**
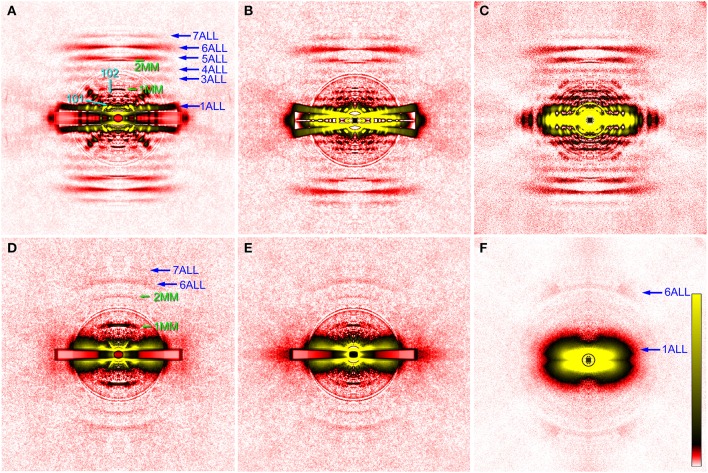
**X-ray diffraction patterns from the glycerinated flight muscle (DLM) of wild-type and mutant *Drosophila***. **(A–C)**, wild type; **(D,E)**, headless myosin mutant (*Mhc^*10*^-Y97*); **(F)** mutant with no myosin heavy chain (*Mhc^10^-*background line). Patterns in **(A–D)** were recorded from single flies, and the pattern in **(F)** is the sum of data from 9 flies. The mounting techniques used are: **(A)**, 2nd (etched plate); **(B,D)**, 3rd (sandwiching with plastic films); **(C,F)**, 1st (round holes); **(D)**, conventional clamping. The four quadrants of the pattern were folded and the background scattering was subtracted as described by Iwamoto et al. (2003, 2010). The circles around the center are due to the correction of absorption by round aluminum masks to attenuate the intense central parts of the pattern. In (**A,B,D**,**E**) a rectangular copper mask was also applied to the equator in which the scattering intensity was strongest. The oblique anomalous reflections in **(A)** come from the remnant of DVM. They are observed in four quadrants because the image has been averaged for the four quadrants. The aberration around the equator in **(B)** is generated in the process of background subtraction because of the mismatch of fiber and copper mask orientations. ALL, actin layer line reflection; MM, myosin meridional reflection. The numbers indicate the order or Miller indices of reflections. Note that only actin-based reflections are observed in **(F)**.

Figure 2 shows some of the diffraction pattern recorded from the indirect flight muscles from wild type and mutant strains of *D. melanogaster*. For wild-type flies, the second technique (using photo-etched stainless steel plates) has so far given the best myofiber orientations (Figure 2A). This diffraction pattern was recorded in rigor (in the absence of ATP), and this sample seems to contain a small remnant of DVM as the pattern contains its equatorial reflections. Although there was some problem in data processing, good myofiber orientation was also obtained with the third technique, i.e., to sandwich the fibers with plastic films (Figure 2B). The fiber orientation was worse with the first technique (Figure 2C), and the conventional (most labor-intensive) technique of clamping both ends of isolated fibers gave totally unsatisfactory results (data not shown). The recorded diffraction pattern is fundamentally similar to that from flight muscle fibers of different insect species (e.g., Tregear et al., 1998; Bekyarova et al., 2008). The lower-angle layer line reflections are finely lattice-sampled, and unlike in relaxed or activated fibers, the 4th–7th actin layer lines (they have continuous intensities along the layer) are clearly visible, indicating that myosin heads are stereospecifically bound to actin following its periodicity.

As for the innermost reflection spots on the 1st and 2nd layer lines, i.e., the 101 and 102 reflections (cyan arrows in Figure 2), the 101 is much more intense than 102, in agreement with that for giant waterbug, *Lethocerus* (Tregear et al., 1998). These reflections are considered to arise from the helical arrangement of troponin complexes around a thick filament, and are known to change their intensities upon stretch activation. Before stretch activation, the 101 is stronger than 102, and vice versa after stretch activation (Tregear et al., 1998; Dickinson et al., 2005; Bekyarova et al., 2008; Iwamoto et al., 2010; Perz-Edwards et al., 2011). This is because a stretch induces stereospecific attachment of myosin heads to the actin target zone located midway between the two neighboring troponin complexes on a thin filament, and these heads negatively interfere with the basic 38.7-nm periodicity of troponin complexes. In rigor, the 101 is intensified again, because excessive myosin binding to the actin target zones restores the 38.7-nm periodicity (Tregear et al., 1998). Together with the reciprocal behavior of these reflections in live flies (Dickinson et al., 2005), the present observations suggest that the numbers of attached myosin heads are similar in *Drosophila* and *Lethocerus* flight muscles, either in active or in rigor states.

The diffraction pattern from the wild-type indirect flight muscle also shows clear myosin meridional reflections based on the 14.5-nm repeat (green arrows in Figure 2). The intensity of the 1st meridional reflection is spread along the equator, in contrast to that of other insects which has a sharper peak right on the meridian (Bekyarova et al., 2008; Iwamoto, 2009). Although the peak splitting is not clear, the present observation agrees with the idea that the thick filaments form a super lattice with a larger lattice constant in *Drosophila* (Squire et al., 2006).

### Diffraction patterns recorded from the headless myosin mutant

The indirect flight muscles of flies with myosin mutations, either myosin-null or headless, have structural defects (Cripps et al., 1999) and are more fragile than those from wild-type flies. Therefore, slicing with vibrating blades often resulted in unsatisfactory processing. The diffraction patterns from the headless mutant as shown in Figure 2 were recorded from either clamped (Figure 2D) or sandwiched (the 3rd procedure, Figure 2E) flight muscle fibers. The two techniques gave equivalent results.

**Figure 3:**
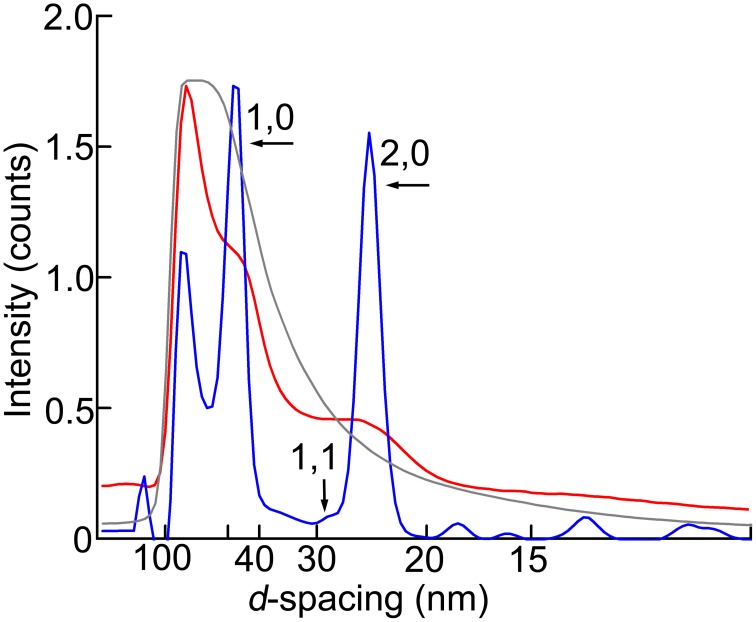
**Intensity profiles of the equatorial reflection**. Blue, wild type; red, headless myosin mutant (*Mhc^*10*^-Y97*); gray, mutant with no myosin heavy chain (*Mhc^10^-*background line). The maximal intensity counts (digitized bits of analog-to-digital converter output) are 2.0 × 10^6^ for wild type, 2.0 × 10^5^ for *Mhc^*10*^-Y97*, and 2.8 × 10^7^ for the *Mhc^10^-*background line.

Compared with the wild type, the pattern from the *Mhc^*10*^*-*Y97* (headless myosin) flight muscle is much more featureless, indicating that the structure is disorganized. Lattice sampling of layer-line reflections are not recognized. However, the 6th and 7th actin layer line reflections are clearly visible. The arcing of these reflections indicates that actin filaments are not well oriented in this mutant. Although it is not clear from the pattern, equatorial reflections are present as their intensity profiles are shown in Figure 3 (red). This indicates that the hexagonal lattice arrangement of myofilaments is maintained in this mutant.

Most notable in the pattern from the *Mhc^*10*^-Y97* indirect flight muscle is the very strong 1st myosin meridional reflection at 14.5 nm^−1^. The 2nd myosin meridional reflection at 7.2 nm^−1^ is also clearly visible. In the diffraction pattern from the *Mhc^*10*^*-background line (missing the entire myosin heavy chain), no meridional reflection was observed at 14.5 nm^−1^ (Figure 2F). This pattern was taken by using the first procedure. In this pattern, the strong 1st actin layer line and the weaker 6th actin layer line are observed. The latter is strongly arced, indicating that the actin filament orientation is less organized in the absence of myosin filaments.

**Figure 4:**
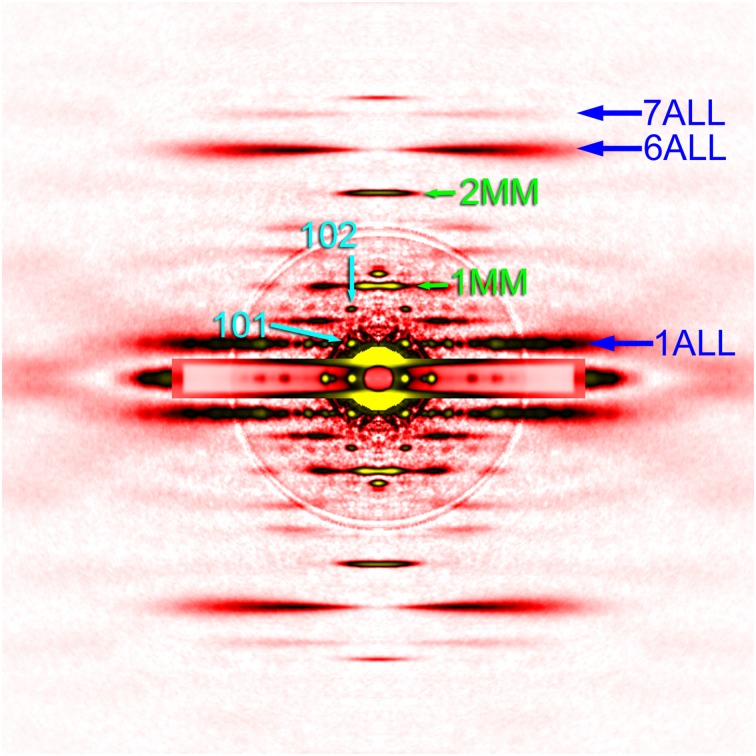
**Diffraction pattern from the flight muscle of a horse fly**. The pattern was recorded in the presence of ATP (relaxing condition). For labels see legend to Figure 2.

To make quantitative comparisons, the integrated intensity of the 1st myosin meridional reflection was normalized to that of the 6th actin layer line reflection, which is often used as an internal standard to compare intensities of various reflections (e.g., Iwamoto, 2009). Its intensity was 76 ± 12% (mean ± S.D., *n* = 5) of that of the 6th actin layer line. Although the patterns were recorded in the absence of ATP, the *Mhc^*10*^-Y97* myosin cannot form rigor linkage to the thin filaments, and therefore the values should be compared with the values from relaxed wild-type flight muscle fibers. In *Drosophila* it was difficult to obtain an ideally relaxed pattern, probably because of the insufficient supply of ATP due to myosin's high ATPase activity (Swank et al., 2006). Instead, we used flight muscle fibers from a bigger dipteran, *Tabanus trigonus* (a horse fly), to obtain relaxed patterns (Figure 4). We recorded patterns from two sets of flight muscle fibers and obtained the intensity ratios of 59 and 64%, which are lower than the values for *Mhc^*10*^-Y97*. Because of the disorder of actin filament orientations in *Mhc^*10*^-Y97*, some of scattering intensities may have been lost to the background scattering, and the intensity of the 6th actin layer line may be underestimated. However, it still holds true that the 1st myosin meridional reflection is unexpectedly strong if its major source is the motor domain of the myosin molecule (see Discussion).

### Electron microscopy

**Figure 5:**
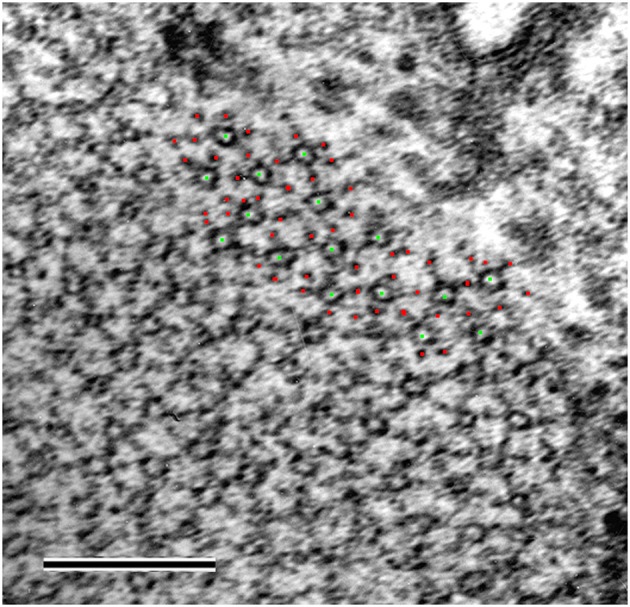
**Electron micrograph of a myofibril in transverse orientation from the headless myosin mutant**. The green and red dots represent the positions of the thick and thin filaments as recognized by eye. Scale bar, 200 nm.

The rationale for using the 6th actin layer line reflection as an internal standard is that the *Mhc^*10*^-Y97* mutant fly retains the same thick-to-thin filament number ratio as in the wild type, in which it is 1:3. To verify this, the cross section of myofibrils of the *Mhc^*10*^-Y97* flight muscle was observed by electron microscopy (Figure 5). Because of the waviness of the myofibrils, clear cross sections of both thick and thin filaments were observed in limited areas. In such an area, the numbers of the recognized thick and thin filaments were 16 (green dots in Figure 5) and 51 (red dots), respectively. This is close to the 1:3 ratio. It therefore appears that the thick-to-thin filament ratio is preserved in the *Mhc^*10*^-Y97* mutant.

**Figure 6:**
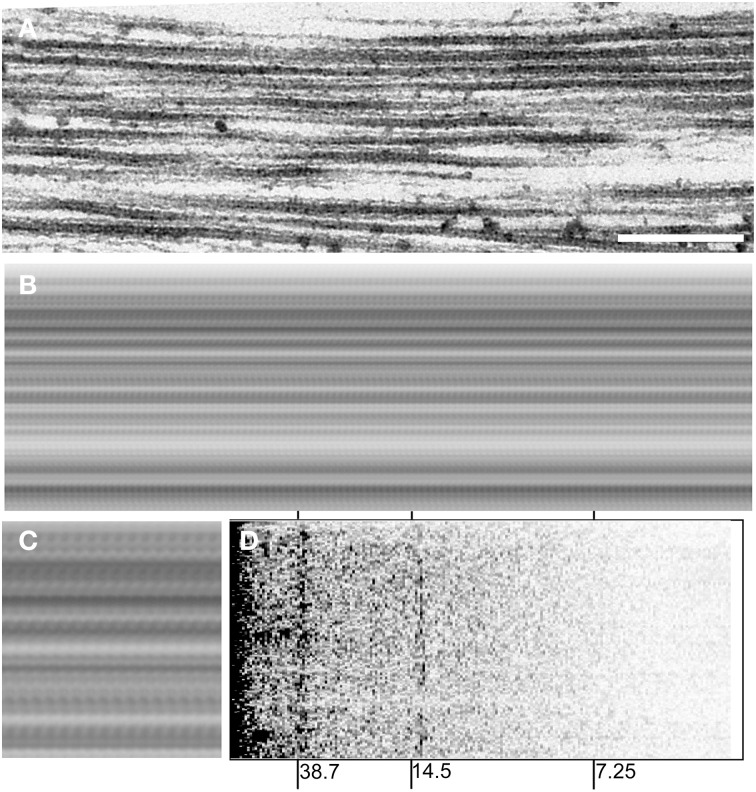
**Electron micrograph of a myofibril in longitudinal orientation from the headless myosin mutant**. **(A)**, a micrograph before processing. Scale bar, 250 nm. **(B)**, a running-averaged picture of the micrograph in **(A)** obtained by superposing the same picture after translating along the filament axis by multiples of 13.8 nm, which gave the best contrast. The difference between 13.8 nm and the expected 14.5 nm could be due to the shrinkage of the specimen and/or uncertainties of magnification of the electron microscope (the 38.7-nm peak is offset to a similar extent). **(C)**, a 2 × magnified view of a part of **(B)**. **(D)**, power spectrum of the micrograph in **(A)**. Note that peaks (darker) appear at around 14.5 nm and 38.7 nm.

The X-ray observation of the strong myosin meridional reflection and the preserved thick-to-thin filament ratio strongly suggest the presence of periodic structures with strong contrast with a 14.5 nm spacing even in the absence of most of the myosin motor domain. We therefore tested whether such periodic structures were visible in the longitudinal sections of myofibrils from the *Mhc^*10*^*-*Y97* indirect flight muscle (Figure 6).

Figure 6A shows one of the longitudinal sections of the mutant myofibril. The picture in Figure 6B is a running-average of the micrograph in Figure 6A, calculated by superposing itself multiple times after translating by a fixed distance. By doing so, features are enhanced if its periodicity coincides with the distance of translation. The summed picture in Figures 6B,C clearly shows an enhanced feature with a periodicity near 14.5 nm. An alternative way of demonstrating the presence of periodic structures is to calculate a power spectrum of the micrograph, by applying Fourier transformation along the filament axis. The power spectrum calculated from the micrograph in Figure 6A clearly shows a peak at ~14.5 nm along with a strong peak at ~38.7 nm, which arises from troponin and tropomyosin on the thin filament (Figure 6D). These results indicate that there are structures with a 14.5-nm structure that are visible by both X-ray and electron microscopy. If there were intact myosin heads, they would bind to actin target zones (these have a 38.7-nm periodicity) and therefore they would contribute to the 38.7-nm peak, rather than 14.5-nm.

## Discussion

In this paper several methods to record X-ray diffraction patterns from glycerinated flight muscle fibers of *Drosophila* are described, and these methods were applied to the wild-type and the *Mhc^*10*^-Y97* (headless myosin) mutant flies. We have shown that, by the use of intense X-rays generated by the 3rd-generation synchrotron radiation facilities, one can record high-quality 2-dimensional diffraction patterns from glycerinated flight muscle fibers. The most important finding is that intense myosin meridional reflections are recorded from the *Mhc^*10*^-Y97* mutant flies in which most of the myosin motor domain is missing.

### The best mounting method for *Drosophila* flight muscle

For wild-type flies, the best-quality patterns were obtained by the second method, i.e., to glue the thorax to a photo-etched stainless steel plate and to remove excess parts by an ultrasonic-vibrated blade. The merit of this method is that only the DLM remains in the hole with its in-situ length, if the thorax has been glued in a proper position (see Figure 1D3) and the blade is moved along the surface of the plate without a gap (the plate is 0.2-mm thick). In contrast to the two other methods, both sides of the flight muscle fibers are exposed to the surrounding medium, so that this method is most suitable for solution exchange experiments.

This method was successfully applied to wild-type flies, probably because of the physical strength of their flight muscle fibers. Although this method was not successfully applied to the more fragile mutant muscles, it is still a promising mounting method for solution exchange for mutant muscles. Improvements of this method, such as automated controlled blade movements (the blades were moved manually in this work) and/or increasing the physical strength of fibers by soaking in glycerol and slicing at low temperatures, may allow us to apply this method also to mutant flight muscles. The throughput of X-ray recording may be increased by creating stainless-steel strips with multiple photo-etched holes (see Figure 1C) instead of just one as in this study.

### The origin of the reflections based on a 14.5-nm-spacing in the mutant fly

The most important finding in this study is the strong myosin-based meridional reflections (at 14.5 and 7.2 nm^−1^) observed in the *Mhc^*10*^*-*Y97* mutant, in which most of the motor domain is missing except for a part of the lever arm and the regulatory light chain. The 14.5 nm-spaced features are also evident in electron micrographs (Figure 6). The strong myosin meridional reflections are unexpected because it is generally believed that the major source of this reflection is the motor domain of the myosin molecule, while the rod domain may contribute to the 2nd reflection (Huxley et al., 1994; Lombardi et al., 2004).

As mentioned briefly in Results, the intensity of the myosin meridional reflection is compared with that of the 6th actin layer line reflection, which might be underestimated because of the disorder. Indeed, the 6th actin layer line seems to be weaker in the mutant, but it is partly explained by the smaller myofibrillar content in the mutant flight muscle cell as shown by Cripps et al. (1999), Figure 6C. On the other hand, the 1st myosin meridional reflection in the mutant pattern is at least as strong as that in the wild type in rigor, despite that its intensity may also be underestimated for the same reason for the 6th actin layer line. Therefore, it still holds true that the 1st myosin meridional reflection is unexpectedly strong.

The question remains whether there are other proteins on the thick filament with a 14.5-nm periodicity. In vertebrate striated muscles, it is known that myosin binding protein C (MyBP-C) has a 42.9-nm periodicity (Rome et al., 1973), and its 3rd-order reflection is expected to overlap with the 1st myosin meridional reflection. MyBP-C is absent from insect flight muscle, but instead, a myosin binding protein flightin has been reported (Vigoreaux et al., 1993). However, the molecular weight of flightin is only 20 kDa and it is unlikely that it contributes significantly to the intensity of the meridional reflection at 14.5 nm^−1^. Another possibility is the remaining myosin lever arm region containing the regulatory light chain (RLC). The RLC has an N-terminal extension that has been suggested to serve in parallel to the motor domain as a link to the thin filament (Moore et al., 2000; Miller et al., 2011). This is similar to the observation that the essential light chain extension of vertebrates appears to bind to actin within thin filaments (Miller et al., 2005; Lowey et al., 2007). If this extension does not preferentially bind to the actin target zone but simply splays out radially, it could strengthen the 1st myosin meridional reflection to some extent. However, the extension is only 50 residues long and again it is unlikely to contribute significantly to the intensity. In fact, removal of the *Drosophila* RLC extension does not diminish the intensity of the 1st myosin meridional reflection (Farman et al., 2009). Insect muscles are known to contain paramyosin, and it may also contribute to some extent to the 1st myosin meridional reflection. Again, however, the paramyosin content in *Drosophila* flight muscle is small (myosin:paramyosin = 34:1; Vinós et al., 1991), and its structural resemblance to the rod portion of myosin makes it unlikely that paramyosin contributes significantly to the 1st myosin meridional reflection.

From the considerations above, we are currently forced to consider that the strong 14.5 nm-spaced feature arise from the truncated myosin heavy chain and the regulatory light chain. It is generally believed that the rod portions of two myosin heavy chains form a coiled-coil structure to form a dimer, and their periodic clusters of charged residues allow the dimers to assemble into thick filaments with a stagger of 14.5 nm (McLachlan and Karn, 1982). There could be a different extent of deviation from the ideal coiled-coil structure between the charged and non-charged regions, and it could contribute to the intensity of the 1st myosin meridional reflection. The present results could indicate that the contribution of such a structural deviation to the 1st myosin meridional reflection is substantial. Conclusive explanations cannot be given from the present results alone, and further experiments are needed to address this issue. These experiments include the creation of a double mutant with (*Mhc^*10*^*-*Y97*) and a knockout of either flightin or RLC or both. It is also important to refine the present method for X-ray diffraction recording to ensure better myofibrillar orientations; it would reduce the ambiguity arising from the poorer filament orientation of actin as compared with myosin.

## Conclusion

Here we have demonstrated that the flight muscle of *D. melanogaster* can serve as a model to study the causes and effects of myosin myopathies, and their structural consequences can be monitored by X-ray diffraction. This should be applicable to studying models of myosin-based congenital myopathies (Wang et al., 2012). In the particular case of the myosin headless mutation (*Mhc^*10*^-Y97*), an unexpected observation of the strong feature with a 14.5-nm periodicity prompts us to reinvestigate the origin of the 1st myosin meridional reflection, one of the best-studied X-ray reflections of muscle.

### Conflict of interest statement

The authors declare that the research was conducted in the absence of any commercial or financial relationships that could be construed as a potential conflict of interest.
